# Maternal valproic acid exposure leads to neurogenesis defects and autism-like behaviors in non-human primates

**DOI:** 10.1038/s41398-019-0608-1

**Published:** 2019-10-21

**Authors:** Hui Zhao, Qiqi Wang, Ting Yan, Yu Zhang, Hui-juan Xu, Hao-peng Yu, Zhuchi Tu, Xiangyu Guo, Yong-hui Jiang, Xiao-jiang Li, Huihui Zhou, Yong Q. Zhang

**Affiliations:** 1 State Key Laboratory of Molecular Developmental Biology, Institute of Genetics and Developmental Biology, CAS Center for Excellence in Brain Science and Intelligence Technology, Chinese Academy of Sciences, Beijing, 100101 China; 2 University of Chinese Academy of Sciences, Beijing, 100049 China; 3 Shenzhen Institutes of Advanced Technology, Chinese Academy of Sciences, Shenzhen, 518055 China; 4 Guangdong Laboratory Animals Monitoring Institute, Guangdong Provincial Key Laboratory of Laboratory Animals, Guangzhou, 510663 China; 5 West China Biomedical Big Data Center, West China Hospital/West China School of Medicine, Sichuan University, Chengdu, 610041 China; 6 Guangdong-Hongkong-Macau Institute of CNS Regeneration, Ministry of Education CNS Regeneration Collaborative Joint Laboratory, Jinan University, Guangzhou, 510632 China; 7 Department of Pediatrics and Department of Neurobiology, Duke University, Durham, NC 27710 USA; 8 Department of Human Genetics, Emory University School of Medicine, Atlanta, GA 30322 USA; 9 Present Address: Key Laboratory of Regenerative Biology, South China Institute for Stem Cell Biology and Regenerative Medicine, Guangzhou Institutes of Biomedicine and Health, Chinese Academy of Sciences, Guangzhou, 510530 China

**Keywords:** Molecular neuroscience, Autism spectrum disorders

## Abstract

Despite the substantial progress made in identifying genetic defects in autism spectrum disorder (ASD), the etiology for majority of ASD individuals remains elusive. Maternal exposure to valproic acid (VPA), a commonly prescribed antiepileptic drug during pregnancy in human, has long been considered a risk factor to contribute to ASD susceptibility in offspring from epidemiological studies in humans. The similar exposures in murine models have provided tentative evidence to support the finding from human epidemiology. However, the apparent difference between rodent and human poses a significant challenge to extrapolate the findings from rodent models to humans. Here we report for the first time the neurodevelopmental and behavioral outcomes of maternal VPA exposure in non-human primates. Monkey offspring from the early maternal VPA exposure have significantly reduced NeuN-positive mature neurons in prefrontal cortex (PFC) and cerebellum and the Ki67-positive proliferating neuronal precursors in the cerebellar external granular layer, but increased GFAP-positive astrocytes in PFC. Transcriptome analyses revealed that maternal VPA exposure disrupted the expression of genes associated with neurodevelopment in embryonic brain in offspring. VPA-exposed juvenile offspring have variable presentations of impaired social interaction, pronounced stereotypies, and more attention on nonsocial stimuli by eye tracking analysis. Our findings in non-human primates provide the best evidence so far to support causal link between maternal VPA exposure and neurodevelopmental defects and ASD susceptibility in humans.

## Introduction

Autism spectrum disorder (ASD) is a group of neurodevelopmental abnormalities characterized by persistent impairment in social communication and interaction, and restricted, repetitive patterns of behaviors, interests, or activities^1^. The prevalence of ASD increased steadily and significantly from one in 150 children during 2000–2002 to one in 59 in 2014 in the United States^2^. However, the etiology for the majority of ASD cases remains unclear, while 20–25% patients having a clear genetic determinant^3,4^. Genetic mutations in genes encoding the proteins involving synaptic development and epigenetic machinery are two major classes of the genes from recent autism genomics studies. Recent studies point to the contribution of non-genetic factors during pregnancy to the development of ASD, such as viral infection, autoimmune diseases, and exposure to teratogens and anticonvulsants such as valproic acid (VPA)^5^. VPA is a widely prescribed antiepileptic drug, but its use during pregnancy may have adverse effects on the offspring. For instance, impaired cognitive function and behavioral abnormalities have been reported in children after fetal exposure to antiepileptic drugs including VPA^6,7^. Several epidemiological studies have linked VPA usage during pregnancy to higher risks of ASD^8–10^, attention-deficit/hyperactivity disorder^11^, and a wide spectrum of malformations known as fetal valproate syndrome^12,13^. However, clinical studies are constrained by many factors to manipulate the system in order to establish causality, and hard to explore the molecular and cellular mechanisms of ASD.

Epigenetic dysregulation is an attractive hypothesis for ASD etiology, especially considering an alarming increase in the prevalence of ASD^14^. Recently, a histone-acetylome-wide association study revealed a characteristic histone acetylation signature in the brain of ASD patients, providing direct evidence for histone modifications, especially acetylation, in ASD-related neuropathology^15^. One known pharmacological action of VPA is inhibition of histone deacetylase (HDAC), and thus VPA might increase the risk of ASD through an epigenetic mechanism^16,17^.

Rodent models that recapitulate certain defects caused by fetal VPA exposure in humans have been established and described. The first VPA-induced rat model was generated with a single intraperitoneal injection to pregnant dams around the time of neural tube closure^18^. These VPA model rats exhibit lower exploratory activity, impaired social behavior, and increased repetitive movements (stereotypies), which are considered autism-related features^19^. Prenatal VPA exposure also enhances NMDA receptor-mediated synaptic plasticity^20^, and disrupts the normal excitatory-inhibitory shift of GABAergic currents during postnatal development^21^, changes that may help explain the emergence of autism-like behaviors. Mice exposed to VPA in utero also exhibit impaired cognitive function in adults, which is related to decreased hippocampal neurogenesis^22^. The ectopic localization of newborn neurons in the hippocampus increases seizure susceptibility in prenatally VPA-exposed adult mice^23^.

Despite many insights derived from rodent models, whether these neuronal defects are conserved between rodents and primates remains unknown. Non-human primates have the potential to validate the knowledge gained from rodent models and provide possible explanations for the effects of in utero VPA on humans^24,25^. Although rodent models are widely used to study human psychiatric disorders, the differences in brain anatomy and behavioral repertoires between rodents and humans preclude simple extrapolation^24–26^. Non-human primates have a much closer evolutional relationship with humans and thus are believed to have greater potential for translational research. For instance, monkeys are widely used for pharmacokinetic and toxicology studies preceding clinical trials^27^. In fact, the adverse effects of VPA have been examined in pregnant rhesus monkeys^28^. Craniofacial and skeletal defects were found in embryos from mothers treated at the dose that is 10 × human clinical dose (common dosage for children 20 mg/kg/day), while 100% of embryos were nonviable when mothers were treated at 30 × clinical dose^28^. However, the effect of VPA on brain development and behaviors in primates has not been addressed.

In this study, we investigated the molecular, cellular, and behavioral changes in cynomolgus monkeys (*Macaca fascicularis*) after early exposure to VPA in utero. We examined changes in gene expression and cellular development in the fetal brain, and aberrant behaviors in VPA-treated juvenile monkeys. Prominent neurogenesis defects and altered gene expression levels were found in the VPA-treated brain. The surviving juvenile offspring exhibited pronounced stereotypies and social interaction defects. Eye-tracking experiments revealed longer fixation towards nonsocial scenes by VPA-treated animals. The neuronal and behavioral defects found in prenatally VPA-exposed monkeys provide novel insights into the pathologies associated with VPA exposure in humans.

## Materials and methods

### Animals and drug administration

Healthy adult cynomolgus monkeys (*Macaca fascicularis*) were housed in cages at Guangdong Blooming-Spring Biological Technology Development Co., Ltd. All animals were housed in a controlled environment (room temperature at 25 °C, humidity 40–70%) with 10–h light/14–h dark cycle (lights on at 0800 hours). All animals were given regular diet twice a day with tap water *ad libitum* and were fed fruits and vegetables once daily. All animal procedures were approved by the Institutional Animal Care and Use Committee of the Institute of Genetics and Developmental Biology, Chinese Academy of Sciences (IGDB-2016-IRB-003).

Fifteen healthy and fertile female monkeys (body weight: 4.61 ± 0.26 kg; number of offspring produced: 5.67 ± 0.40; age: 9.33 ± 0.23 years old, data are presented as mean ± s.e.m., *n* = 15) were caged separately (cage size: 0.75 m L × 0.85 m W × 1 m H) for menstrual cycle observation over three months. Individual female was housed with one male (age: 8.75 ± 1.49 years old) for 5 days beginning 2 days before expected ovulation to facilitate mating. The day of ovulation was designated as gestational day (GD) 0. Pregnancy was confirmed in nine female monkeys by ultrasonographic examination (Vevo2100, VisualSonics, Toronto, CA) on GD 19–23 under general anesthesia by intraperitoneal injection with Zoletil 50 (4–6 mg/kg, Virbac S.A., France). Nine age- and gender-matched monkeys from control mothers without anesthesia and VPA injection were selected as controls. The controls were raised in conditions similar to that of VPA-treated group.

Two critical parts of the design are the time window and dosage of VPA exposure. The in utero rats were exposed to VPA once during E11.5–12.5 to induce autistic-like features^18^, a period around neural tube closure (E10–12)^29^. It has been speculated that VPA also disturbs the same stage to cause autism in humans^18,30^. We decided to administrate VPA twice on GD 26 and 29 based on the longer time of neural tube closure of monkey embryos. One group of five pregnant monkeys was intraperitoneally injected with 200 mg/kg NaVPA (Meilunbio, Dalian, China; dissolved in 0.9% sterile saline, pH 7.3), and another group of four pregnant monkeys was treated with 300 mg/kg NaVPA. The dose of 200 mg/kg in monkeys is equivalent to 500 mg/kg in rats^20^. The low pregnancy rate (9/15) and high abortion rate (4/9) in VPA-treated monkeys contributed to a small sample size.

All treated and untreated monkey offspring are listed in Table 1. Infant offspring were raised with their mothers in home cages (0.8 m L × 0.6 m W × 2 m H) and weaned at about 10 months of age. The juveniles were housed in home cages in groups before behavioral tests.

**Table 1:** Information of each animal

Animal	DOB (y.m.d)	Gender	Dose (mg/kg)	GD	I.P. time
**Behavioral assay**					
Treated 1 (t1)	2015.12.8	Female	200	163	GD 26 and 29
Treated 2 (t2)	2016.1.23	Female	200	170	GD 26 and 29
Treated 3 (t3)	2016.3.3	Female	200	176	GD 26 and 29
Treated 4 (t4)	2016.4.23	Female	300	172	GD 26 and 29
Treated 5 (t5)	2016.2.18	Male	300	173	GD 26 and 29
Control 1 (ctl1)	2015.12.16	Female	NT	Full term	NT
Control 2 (ctl2)	2016.1.24	Female	NT	Full term	NT
Control 3 (ctl3)	2016.3.5	Female	NT	Full term	NT
Control 4 (ctl4)	2016.4.25	Female	NT	Full term	NT
Control 5 (ctl5)	2016.2.19	Male	NT	Full term	NT
Treated 6 (t6)	2015.8.9	NK	200	29 (Abortion)	GD 26 and 29
Treated 7 (t7)^#^	2016.3.2	Female	300	160 (Stillbirth)	GD 26 and 29
**Mol. and cell. assay**					
Treated 8 (M1)	2015.12.30	Male	200	166 (Abortion)	GD 26 and 29
Treated 9 (M2)	2016.4.17	Female	300	166 (Abortion)	GD 26 and 29
Ctl1	2017.6.23	Male	NT	Full term abortion	NT
Ctl2	2017.6.24	Female	NT	Full term abortion	NT
Ctl3	2017.6.22	Male	NT	Full term abortion	NT
Ctl4	2016.4.24	Male	NT	Full term abortion	NT

*DOB* date of birth, *NK* not known, *NT* no treatment, *GD* gestational day, full term, gestational day 165 ± 10; # indicates sample not fresh enough for experiment, I.P. intraperitoneal injection

### Immunohistochemistry

For immunostaining, the brains of aborted fetuses and untreated controls were removed and fixed for 48 h in 4% paraformaldehyde. Different brain regions including the PFC and cerebellum were dissected out and paraffin-embedded. Paraffin-embedded tissues were sliced into 4-μm-thick sections. The primary antibodies used in this study are listed in Supplementary Table 1. Samples were incubated with corresponding HRP-conjugated secondary antibodies (anti-mouse or anti-rabbit, 1:1000; Dako, USA). DAB (3, 3'-diaminobenzidine) staining was used for chemiluminescent detection and hematoxylin for nuclear staining. Images were acquired with a Leica SCN400 Slide Scanner (Leica Microsystems).

For cell density analysis, cells within specific areas ( > 0.2 × 0.1 mm^2^) across all layers of PFC were counted manually. The area of positive NeuN staining in the cerebellar internal granular layer and the thickness of the Ki67-positive (proliferating) external granular layer of cerebellum were measured by ImageJ.

### Western blotting

Prefrontal cortex (PFC) was homogenized in RIPA buffer (Hua Xing Bo Chuang, with 1 × protease inhibitor cocktail) on ice. Supernatant protein was separated by SDS-PAGE and transferred onto PVDF membranes (Millipore). Carbonate blot buffer (10 mM NaHCO_3_, 3 mM Na_2_CO_3_, pH 9.9 and 20% methanol) was used for efficient electrophoretic transfer of histones to membranes. The primary antibodies used are listed in Supplementary Table 1. Specific bands were quantified by ImageJ and normalized to α-tubulin expression (the gel loading control).

### RNA preparation and sequencing

Total RNA was extracted from the PFC of VPA-exposed (M1 and M2) and age-matched control monkeys (Ctl1 and Ctl2). Only samples with RNA integrity number (RIN) over 6.8 were used for cDNA library construction. Sequencing was performed on a single lane of an Illumina HiSeq 4000 to produce 150 bp paired-end reads. The clean reads were aligned to the cynomolgus monkey genome (ftp://ftp.ncbi.nlm.nih.gov/genomes/all/GCF/000/364/345/GCF_000364345.1_Macaca_fascicularis_5.0/GCF_000364345.1_Macaca_fascicularis_5.0_genomic.fna.gz) using TopHat2 software (http://ccb.jhu.edu/software/tophat/index.shtml). Normalized transcript abundance was estimated by the expected fragments per kilobase of transcript per million fragments mapped (FPKM) using Cuffnorm (http://cufflinks.cbcb.umd.edu/). We performed three independent replicates from adjacent areas for each animal. Differentially expressed genes (DEGs) between VPA-treated and control monkeys were filtered using the DEseq package (http://www.bioconductor.org/packages/release/bioc/html/DESeq.html). DEGs defined by (1) fold change (FC) > 2 or < 0.5 and (2) false discovery rate (FDR) < 0.01 were grouped into different Kyoto Encyclopedia of Genes and Genomes (KEGG) enrichment pathways.

### Quantitative real-time PCR (qRT-PCR)

Extracted RNA was reverse transcribed using a SuperScript III First Strand cDNA synthesis kit (Invitrogen), followed by qRT-PCR using SYBR Green PCR Master Mix (ABI) on a Real-Time QPCR System (Agilent). The relative mRNA expression levels were analyzed according to the ΔΔCt method^31^. *GAPDH* was used as the reference gene. The genes and primers used for qRT-PCR are listed in Supplementary Tables 2 and 3. For validation of RNA-seq results, we compared the qPCR results with RNA-seq data using Pearson correlation test.

### Behavior analysis

For behavioral analysis, the surviving juvenile monkeys at 17–21 months of age (5 VPA-treated and 5 controls in total; Supplementary Table 4) were re-housed in observation cages (2 m L × 1 m W × 1 m H). All animals were divided into three groups (cage 1–3). In each cage, the VPA-treated monkeys of similar ages were grouped with the age- and sex-matched controls. In cage 3, two animals (ctl2 and t2) from cage 1 were re-used to ensure an equal group size.

Behaviors of all animals were video-recorded for 1 h daily at the same time (1000–1100 hours or 1500–1600 hours) of the day for 5 days within two weeks. A 20-min video in the middle of 1-h recording was selected for analysis. Five 20-min videos collected from 5 days were analyzed for each animal. Each animal wore a colored collar to be identified. We quantified the duration of active and passive social contact, stereotypical behaviors, and environmental exploration from videos using an established ethogram (see Supplementary Table 5). Each video-recording was analyzed by three trained technicians blinded to experimental conditions. An inter-observer reliability > 85% was established before scoring. During the scoring, the technician recorded the frequency and duration of a specific behavior by manually starting and stopping the video.

### Noninvasive eye tracking

Eye-tracking analysis was performed largely following a previously published protocol^32^. Animals (5 VPA-treated and 5 control juvenile monkeys; Supplementary Table 4) were seated in a primate chair with their head fixed by a helmet 57 cm in front of a 27-inch 16:9 computer screen. Gaze positions were sampled at 1000 Hz using an infrared eye-tracker (SensoMotoric). Visual stimulus presentation and data collection were performed using the MonkeyLogic Toolbox of MATLAB (http://www.monkeylogic.net). We presented four types of visual stimuli to investigate animals’ attention and social processing. The four types of stimuli include (1) macaque faces (“face”, *n* = 20 images) (https://visiome.neuroinf.jp/primface), (2) natural complex scenes including monkeys (“social & other”; “social” refers to the monkeys while “other” indicates regions without monkeys, *n* = 25 images), (3) natural complex scenes without monkeys (“nonsocial”, *n* = 25 images), and (4) simple scenes such as grass land (“simple”, *n* = 10 images). Each stimulus was presented for 5 s followed by a 2-s blank gray screen. The image size was 17.8 × 17.8° of visual angle for “face” stimuli, and 17.8 × 13.3° for the remaining three types of stimuli. All animals were tested twice on two different days within one week. Data of five monkeys in each group from 2 days were averaged and each data point represents the results from an individual monkey.

### Statistical analysis

Data analysis was conducted using SPSS IBM 19. Sample sizes were not pre-determined using statistical methods. The normality and variance of data distribution between two groups were analyzed by Kolmogorov–Smirnov test and Levene's test, respectively (*P* > 0.05). Unpaired Student’s *t* test (two-tailed) was performed for all experiments except for RNA-seq data analyses. *P* < 0.05 is considered indicative of significant differences between groups. No data points were excluded for any of the experiments. All data are presented as mean ± s.e.m.

## Results

### Increased abortion risk but largely normal infant development upon in utero VPA treatment

**Fig. 1:**
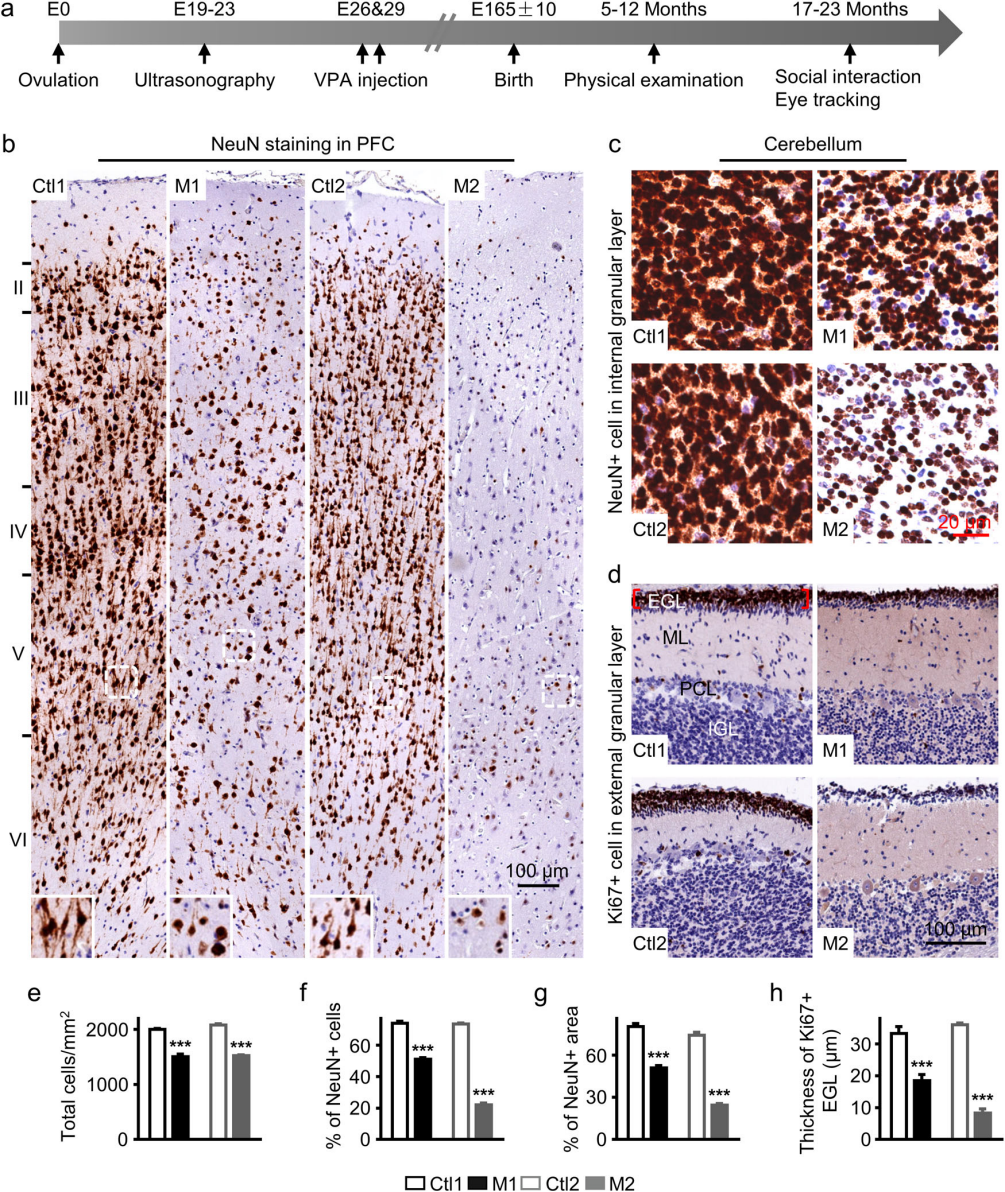
Effects of in utero VPA exposure on cortical and cerebellar development. **a** Diagram of the experiment design. Pregnant monkeys were treated with 200 or 300 mg/kg VPA on G26 and 29. The full-term aborted fetuses were used for molecular and cellular analysis. Physical development, social interactions, and eye tracking were examined in the live offspring. **b** NeuN staining (brown) to label mature neurons in the prefrontal cortex (PFC). Nuclei were stained with hematoxylin (blue). Cell density and the ratio of NeuN-positive neurons appeared substantially reduced in each PFC layer of VPA-treated monkeys compared to age- and sex-matched controls. NeuN-positive cortical pyramidal neurons also showed aberrant dendritic morphology (insets). **c**, **d** Immunostaining for NeuN (brown) to mark mature neurons **c** and Ki67 (brown) to mark proliferating granule cell precursors **d** in cerebellum. Nuclei were stained with hematoxylin (blue). The density of NeuN-positive cells **c** and the thickness of EGL **d** appeared substantially lower in VPA-treated monkey cerebellum. EGL external granular layer; ML molecular layer; PCL Purkinje cell layer, IGL internal granular layer. **e**–**h** Quantitative analysis of total cell density **e**, the percentage of NeuN-positive cells in layers II–VI of the PFC **f**, the percentage of NeuN-positive area in the cerebellar granule cell layer **g**, and the thickness of the region stained by anti-Ki67 **h**. Scale bars: 100 μm for **b** and **d** and 20 μm for **c**; error bars represent s.e.m.; *P < 0.05, **P < 0.01, and ***P < 0.001 by Student’s *t*-test

To investigate the effects of in utero VPA exposure in primates, we injected VPA intraperitoneally during neural tube closure. The detailed experiment schedule is shown in Fig. 1a. It has been reported that rats exposed to VPA during E11.5–12.5, a period around neural tube closure (E10–12), manifest autism-like behaviors^18,19^. Neural tube closure occurs rapidly over a two-day period in rodents, while a comparable process requires approximately one week in primates^33^. Therefore, VPA (200 or 300 mg/kg, double injections) was administered by intraperitoneal injection (i.p.) on GD 26 and 29 (Table 1), during which time neural tube closure is expected to occur in monkey embryos^33^. The dosage for i.p. route used was extrapolated from rat models^19,20^, about 10 times the human therapeutic dose of 20–30 mg/kg/day.

After timed mating, we obtained nine pregnancies verified by ultrasonographic examination. Of these, four (t6, t7, M1 and M2) aborted after VPA administration. Among these abortions, one (t6) occurred on G29 shortly after VPA administration as evidenced by menstrual bleeding, while the other three were full-term abortions. Two fresh embryos, M1 and M2, were collected for further pathological analysis while the other (t7) was stillborn and not fresh enough for further analysis. In addition, we examined developmental and behavioral abnormalities in the five live VPA-treated monkey offspring (t1–t5). These VPA-treated monkeys, as well as age- and sex-matched controls were housed with their own mothers separately until 10 months old. Body weight, head circumference, and chest circumference of VPA-treated and age-matched control monkeys were measured each month starting from five months after birth. Compared to control monkeys, body weight, head circumference, and chest circumference of the VPA-treated monkeys appeared to be comparable to that of the controls (Supplementary Fig. 1).

### Disrupted neurogenesis in the brain of VPA-treated monkeys

To examine whether VPA exposure may result in neuropathological changes, we performed immunochemical analysis with neuronal and glial markers in the brains of two aborted embryos, M1 (male) and M2 (female). NeuN is a neuron-specific nuclear protein commonly used to identify mature neurons, while GFAP is a known marker for astrocytes. We focused on two different brain regions, PFC and cerebellum, to indicate general changes across the primate brain. The layered structure of the PFC was compared between VPA-treated (M1 and M2) and two age- and sex-matched control embryos (Ctl1 with M1 and Ctl2 with M2). At the cellular level, we examined the morphology and number of neurons in the PFC. Typically, the apical dendrites of pyramidal neurons extend vertically above the soma toward the brain surface. In M1, however, the morphology of NeuN-positive cells exhibited smaller or absent dendritic extensions compared to the controls (Fig. 1b). Additionally, we found significantly fewer cells, based on hematoxylin staining, in the PFC of VPA-treated monkeys with largely normal structure of each layer (Fig. 1b, e). The cell density of cortical layers II to VI was significantly lower in VPA-treated monkeys than controls (2000 ± 20.69 cells/mm^2^ for Ctl1 vs. 1506.3 ± 46.45 cells/mm^2^ for M1, *P* < 0.001; 2079.2 ± 27.53 cells/mm^2^ for Ctl2 vs. 1525 ± 16.32 cells/mm^2^ for M2, *P* < 0.001; *n* = 4 regions for each layer; Fig. 1b, e). We further analyzed the percentage of NeuN-positive cells in layers II–VI of PFC (Fig. 1b, f) and the percentage of NeuN-positive area in the cerebellar granular layer (Fig. 1c, g). Both VPA-exposed brains exhibited a lower percentage of NeuN-positive cells in the PFC compared to controls (73.94% ± 1.42% NeuN^+^ cells/total cell counts for Ctl1 vs. 51.15% ± 0.95% for M1, *P* < 0.001; 73.44% ± 0.73% for Ctl2 vs. 22.1% ± 1.14% for M2, *P* < 0.001; *n* = 6 regions for each layer; Fig. 1b, f), as well as a smaller NeuN-positive area in the cerebellar internal granular layer (80.33% ± 2.11% area for Ctl1 vs. 50.85% ± 1.66% for M1, *P* < 0.001; 74.05% ± 2.22% for Ctl2 vs. 24.45% ± 1.19% for M2, *P* < 0.001; *n* = 6 regions for each individual; Fig. 1c, g). In contrast, the number of GFAP-positive astrocytes per square millimeter was higher in the PFC of M1 and M2 compared to their matched controls (Supplementary Fig. 2a, b).

The significantly lower cell density in the PFC and cerebellum of VPA-exposed brain suggests that neuronal production may be reduced. To study the effects of VPA exposure on neural cell proliferation directly, we stained the stem cell layer of cerebellum with the cell proliferation marker Ki67. The external granular layer (EGL) covering the cerebellar surface is mainly composed of granule cell precursors in the embryonic brain^34^. The Ki67-positive EGL was significantly thinner in VPA-exposed brains compared to controls (33.32 ± 1.08 μm for Ctl1 vs. 18.47 ± 0.97 μm for M1, *P* *<* 0.001; 36.03 ± 0.29 μm for Ctl2 vs. 8.31 ± 0.64 μm for M2, *P* < 0.001; *n* = 12 measurements from each individual; Fig. 1d, h). These results together demonstrate that prenatal VPA exposure disrupts normal neurogenesis in PFC and cerebellum of the monkey brain.

### Prenatal VPA alters the expression of neural markers and synaptic proteins in the PFC

**Fig. 2:**
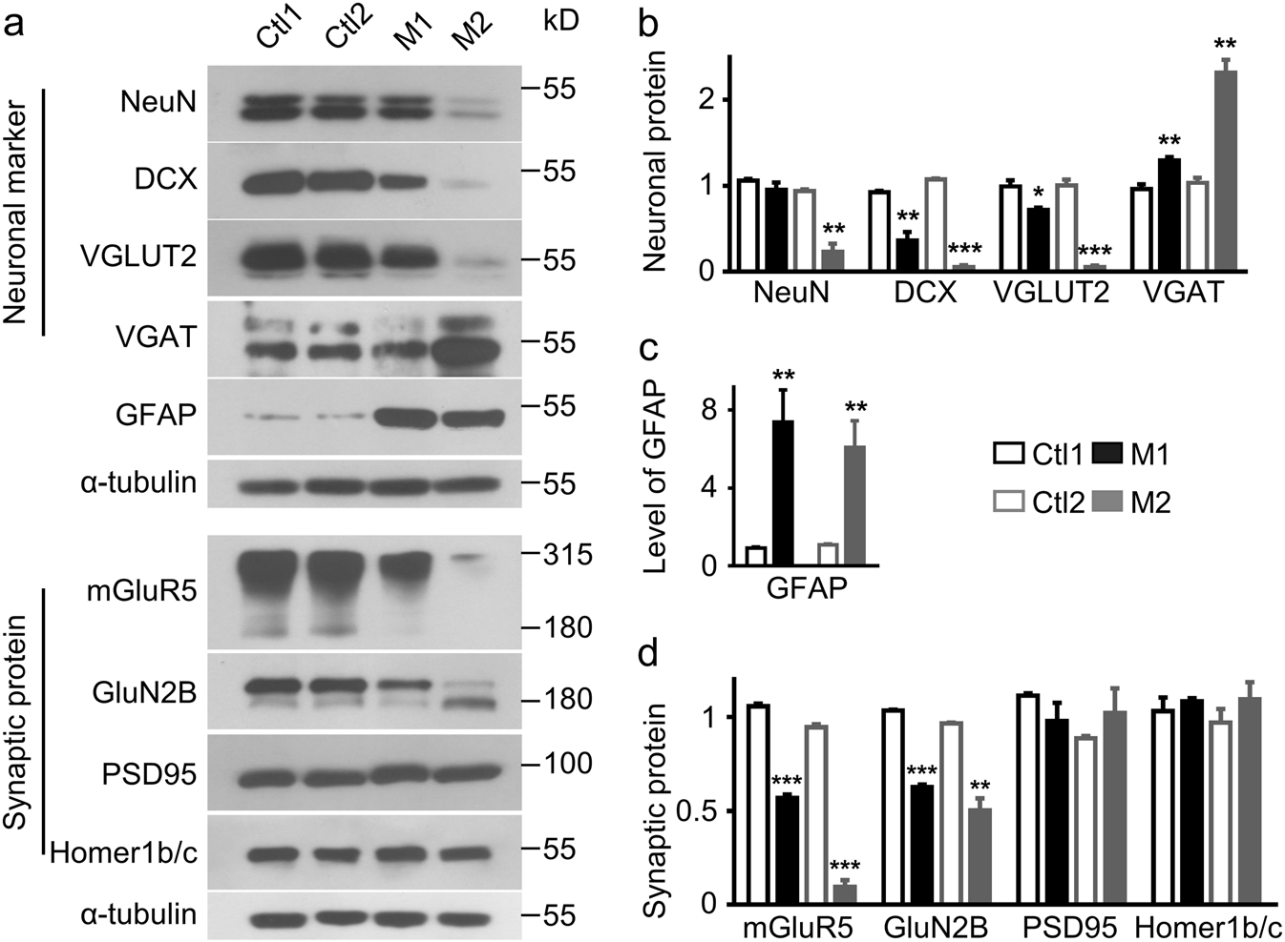
Altered expression levels of neuronal and glial markers and synaptic proteins in VPA-treated monkey brain. **a** Reduced expression of neuronal markers (VGLUT2, DCX, and NeuN) and glutamate receptors (mGluR5 and GluN2B), but increased expression of the GABAergic marker VGAT and glial marker GFAP in the PFC of VPA-treated monkey brain. Ctl1 and Ctl2 are age- and sex-matched controls. **b**–**d** Quantification of the relative expression levels of neuronal markers **b**, the glial marker GFAP **c**, and synaptic proteins **d**. Western blotting of independently prepared samples was repeated at least three times; error bars represent s.e.m.; *P < 0.05, **P < 0.01, and ***P < 0.001 by Student’s *t*-test

Prenatal VPA exposure has been reported to alter neural protein expressions in rodents^20,35^. We therefore compared the expression levels of neuronal markers and synaptic proteins in the PFC of VPA-treated and control monkeys by western blotting (Fig. 2a). VPA exposure altered the expression of neuronal and glial markers, consistent with results of immunohistochemistry (Fig. 1b and Supplementary Fig. 2a). In utero VPA reduced the expression levels of both the mature neuron marker NeuN (M1, 89.99% of Ctl1, *P* = 0.2939; M2, 25.07% of Ctl2, *P* = 0.0019; *n* = 3) and the immature neuron marker doublecortin (DCX) (M1, 39.35% of Ctl1, *P* = 0.0049; M2, 5.34% of Ctl2, *P* < 0.001; *n* = 3), but increased GFAP expression (M1, 809.05% of Ctl1, *P* = 0.0044; M2, 561.2% of Ctl2, *P* = 0.0065; *n* = 5) (Fig. 2a–c), again suggesting that VPA inhibits neurogenesis and promotes astrocyte production or activation during embryo development.

While there were no differences in the levels of PSD95 and Homer1b/c between VPA-treated and control samples (Fig. 2a, d), VPA-treated PFC expressed significantly lower levels of metabotropic glutamate receptor 5 (mGluR5) (M1, 53.74% of Ctl1, *P* *<* 0.001; M2, 10.07% of Ctl2, *P* < 0.001; *n* = 3), NMDA receptor subunit GluN2B (M1, 60.5% of Ctl1, *P* < 0.001; M2, 52% of Ctl2, *P* = 0.0021; *n* = 3) (Fig. 2a, d). We note that changes in the expression levels of NeuN, DCX, mGluR5 and GluN2B, but not GFAP, were greater in M2, which was exposed to a higher dose of VPA than M1 (300 vs. 200 mg/kg).

In addition to the alterations in the expression of neural markers and a subset of glutamate receptors, we found opposite changes in protein levels of glutamatergic and GABAergic markers in VPA-treated brains. Vesicular glutamate transporter 2 (VGLUT2), a marker for glutamatergic synapses, was significantly lower in the PFC of VPA-treated monkeys (M1, 72.57% of Ctl1, *P* = 0.0215; M2, 5.63% of Ctl2, *P* < 0.001; *n* = 3; Fig. 2a, b). Conversely, vesicular GABA transporter (VGAT), a GABAergic synapse marker, was significantly higher in the PFC of VPA-treated monkeys (M1, 134.63% of Ctl1, *P* = 0.0087; M2, 223.34% of Ctl2, *P* = 0.0014; *n* = 3; Fig. 2a, b). Notably, the embryo exposed to the higher VPA dose (M2) exhibited more prominent changes in the protein level of VGLUT2 and VGAT, similar to the changes of the neuronal markers NeuN and DCX. These results suggest that in utero VPA exposure may alter the balance between excitatory and inhibitory (E/I) synapses in monkey PFC.

### Altered transcriptome in VPA-treated monkey brain

**Fig. 3:**
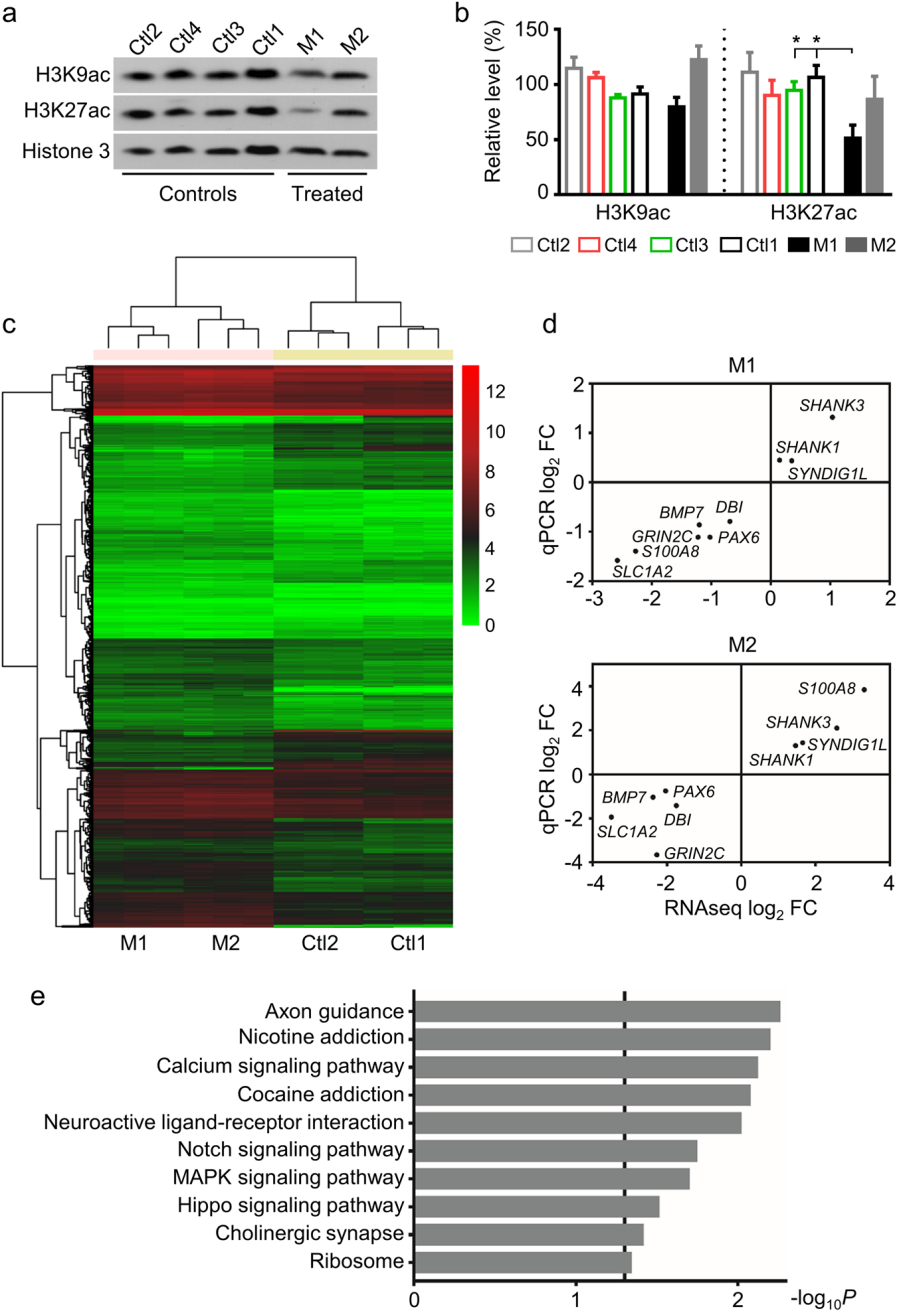
RNA-seq analysis of VPA-treated monkey prefrontal cortex shows large-scale alterations in the transcriptome. **a** Representative blots of acetylated histone H3 at K9 and K27 in PFC of VPA-treated and control monkeys. **b** Statistical analysis shows reduced expression of H3K27ac in M1 PFC. Error bars represent s.e.m.; *P < 0.05 by Student’s *t*-test. **c** Heat map representation of differentially expressed genes (DEGs) in VPA-treated monkeys compared to controls. DEGs were distinguished by false discovery rate (FDR) ≤ 0.01 and fold change (FD) ≥ 2 or ≤ 0.5. The columns indicate different samples with three replicates and the rows represent the 1722 DEGs identified between VPA-treated and control monkeys clustered by similar expression patterns. The color denotes the expression levels of different genes as indicated by log_2_ (FPKM + 1). FPKM, expected fragments per kilobase of transcript per million fragments mapped. **d** qRT-PCR validation of the RNA-seq results in a VPA-treated male (M1) and a female (M2) monkey. *GAPDH* was used as the reference gene. The values in both axes denote log_2_ (FC) in expression. Pearson's correlation coefficient, *r* = 0.9509, *P* < 0.0001 for M1 and *r* = 0.9242, *P* = 0.0004 for M2. **e** KEGG (Kyoto Encyclopedia of Genes and Genomes) enrichment analysis of the DEGs in response to VPA exposure. Among 1,722 DEGs, 882 are functionally mapped into different KEGG pathways. The vertical line indicates *P* = 0.05 (−log_10_ *P* = 1.301). *P* value represents Benjamini–Hotchberg correction for multiple tests

While the mechanism of anticonvulsant effect for VPA is not completely understood, one of the known pharmacological actions of VPA is inhibition of HDAC^16^. Extensive genetic studies of ASD have uncovered the etiological role of epigenetic dysregulation in ASD^36,37^. We therefore examined a potential effect of VPA on histone acetylation in the monkey brain in two aborted fetuses. In contrast to the increased level of acetylated histone shortly (a few days) after VPA treatment reported in literature^38,39^, we found a significantly reduced level of acetylation of H3K27 (H3K27ac) in the PFC of M1 (M1, 48.20% of Ctl1; *P* = 0.0115; *n* = 5; Fig. 3a, b), but no significant change in acetylation of H3K9 (H3K9ac) in the PFC of both M1 and M2 ~130 days after VPA exposure.

VPA could have widespread effects, direct or indirect, on gene expression by affecting histone acetylation levels^16,17^. Therefore, we performed RNA-seq analysis in the PFC of two VPA-treated embryos (M1 and M2) and two age- and sex-matched control embryos (Ctl1 and Ctl2). We analyzed three independent samples from each animal for biological repeats. The clean reads were aligned to the genome of crab eating macaque (version: Mfa5.0). In total 15,095 expressed genes were retrieved across all samples. Genes expressed in two VPA-treated monkeys were compared to that of two control monkeys, and we focused on genes with shared changes in two VPA-treated monkeys to exclude the effect of sex difference and dosage difference. Gene expression analysis identified 1722 differentially expressed genes (DEGs) in both VPA-treated monkey brains (FDR ≤ 0.01 and log_2_ FC ≥ 1 or log_2_ FC ≤ −1; Fig. 3c). Among these, 1187 were upregulated and 535 were downregulated (see Supplementary Table 6 for gene lists). We selected 9 genes that were either having large fold changes in expression or functioning in neurodevelopmental processes (Supplementary Table 2) from RNA-seq for validation analysis using qRT-PCR. Comparison of gene expression levels obtained from RNA-seq and qRT-PCR showed a highly significant Pearson correlation coefficient (r) (M1: *r* = 0.9509, *P* < 0.0001; M2: *r* = 0.9242, *P* = 0.0004; Fig. 3d). Neuronal marker DCX was significantly decreased in RNA-seq analysis, consisting with the Western results in Fig. 2c. To identify the functional pathways involving these DEGs, we conducted KEGG enrichment analysis. Among the 1722 DEGs, 882 are functionally annotated by the KEGG database. The top 10 enriched pathways are shown in Fig. 3e. The most significantly disrupted pathway in both VPA-treated brains was axon guidance (ko04360), including guidance cues (e.g., netrins and semaphorins) that regulate actin cytoskeleton, thereby affecting neuronal network formation^40^. In the calcium signaling pathway (ko04020), *CaMKII* (calcium/calmodulin-dependent protein kinase II), critically involved in learning and memory, was significantly upregulated (Supplementary Table 6), consistent with a previous finding in a VPA rat model^20^. In summary, the top KEGG pathways induced by VPA treatment are involved in neuronal development and signaling.

### Impaired social interaction and stereotypies in VPA-treated monkeys

**Fig. 4:**
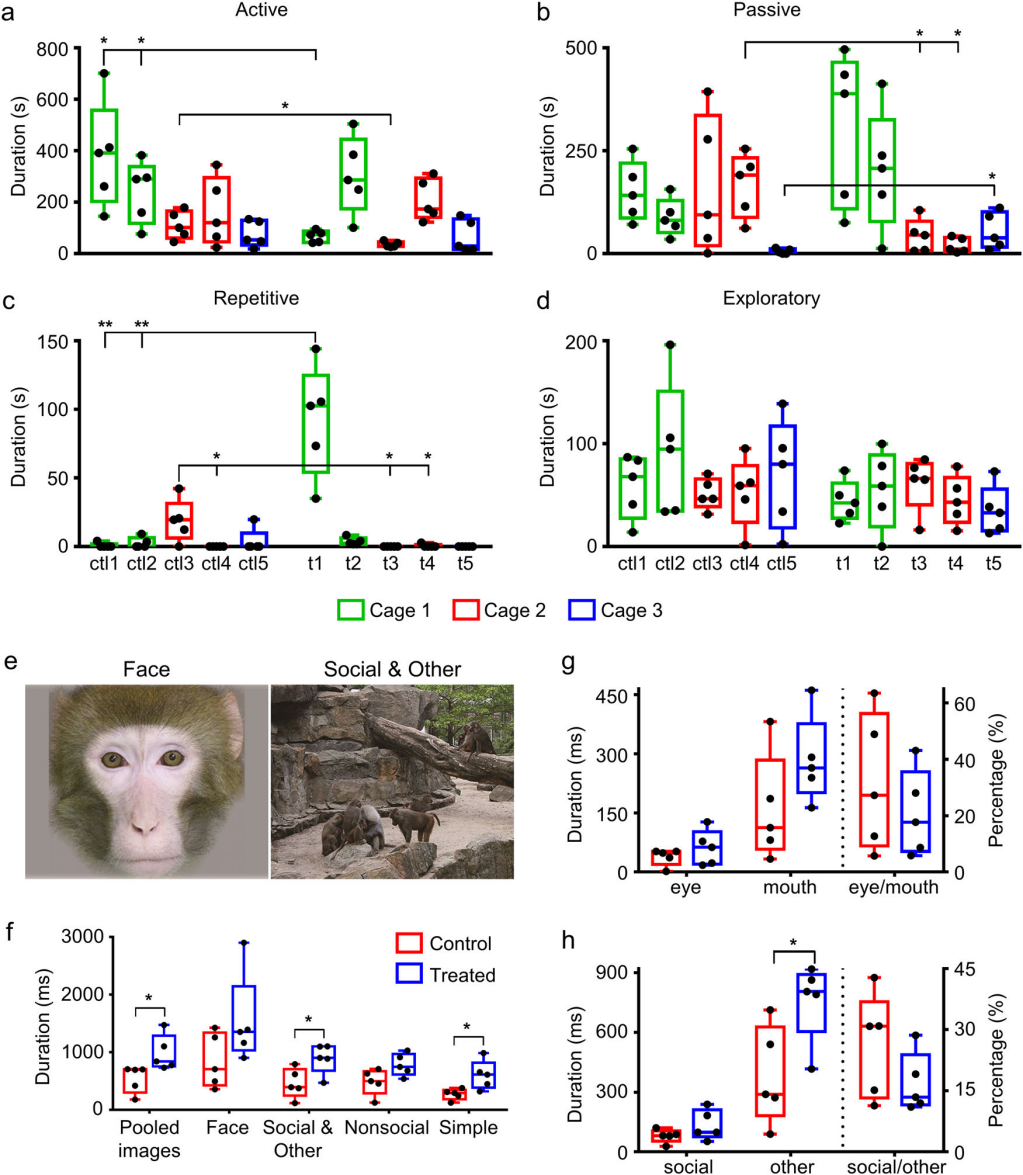
Autism-related behavioral abnormalities and altered eye gaze toward social stimuli in VPA-treated monkeys. **a–d** Active/passive social interaction durations, defined by the average time a monkey initiated **a** or received **b** interaction with any other monkey in the group during a 20-min period. Stereotypical **c** and exploratory **d** behaviors depicting the durations of repetitive circling and exploration towards the surroundings, respectively. *n* = 5 for VPA-treated and control monkeys each. Three monkeys (t1, t2, and t3) were exposed to 200 mg/kg VPA twice on GD 26 and 29. The other two monkeys (t4 and t5) were exposed to 300 mg/kg VPA twice on GD 26 and 29. Each boxplot represents the data of one monkey and each dot depicts the data recorded daily from one monkey. Different colors indicate different cages. **e** Examples of representative stimuli used in the eye-tracking test: “face” (n = 20 different images) and “social & other” (natural complex scene with monkeys, *n* = 25 images). **f** The average time spent on each stimulus of 80 total images including “face”, “social & other”, “nonsocial”, and “simple”. VPA-treated monkeys spent significantly more time on “social & other” and “simple” stimuli. **g** Fixation duration directed at the region of “eye” or “mouth” of the image, and the ratio of duration on “eye” to that of “mouth”. **h** Fixation duration directed at the region of “social” or “other” of the image, and the ratio of duration on “social” to that of “other”. Each dot represents the data from one monkey. Error bars represent s.e.m., *P < 0.05 and **P < 0.01 by Student’s *t*-test

We also analyzed social behaviors in VPA-treated monkeys (t1–t5), as defects in social interaction are reported in VPA rodent models^19^. All the live monkeys (5 VPA-treated and 5 untreated controls, Supplementary Table 4) were habituated in the observation cages before the social test. Behaviors of each VPA-treated monkey were quantitatively compared with individual controls within the same cage. The social behaviors of a tested individual were categorized as active or passive based on 1-h daily observation for 5 days, but a 20-min video in the middle of 1-h recording from each day was statistically analyzed. The average time of VPA-treated monkeys (t1 and t3) actively interacting with other monkeys was significantly lower than that of the controls within the same cage (67.32 ± 10.21 s for t1 vs. 382.5 ± 93.16 s for ctl1 and 240.6 ± 54.29 s for ctl2, *P* = 0.01 and 0.032, respectively; 35.45 ± 4.68 s for t3 vs. 110.7 ± 24.36 s for ctl3, *P* = 0.035; Fig. 4a). The VPA-treated monkeys (t3 and t4) also received less passive social interaction from other monkeys within the same cage (43.53 ± 17.99 s for t3 and 19.82 ± 8.16 s for t4 vs. 166.5 ± 34.6 s for ctl4, *P* = 0.014 and 0.012, respectively), while t5 received more social interactions than the control monkey (5.468 ± 2.76 s for ctl5 vs. 54.4 ± 19.88 s for t5, *P* = 0.041; Fig. 4b). We note that one control monkey (ctl3) displayed mild but significant stereotypy compared with the other three monkeys within cage 2 (Fig. 4c). Importantly, one VPA-treated monkey (t1) exhibited significantly more repetitive walking compared to controls within cage 1 (92.19 ± 18.2 s for t1 vs. 0.84 ± 0.84 s for ctl1 and 2.55 ± 1.79 s for ctl2, *P* = 0.007 and 0.008, respectively; Fig. 4c and Supplementary Video). However, the time spent on exploration of the surroundings was comparable between VPA-treated and control monkeys within all three cages (Fig. 4d). Therefore, VPA exposure disturbs social behaviors in some juvenile monkeys, while exploratory behaviors appear unaffected.

### Abnormal visual attention in VPA-treated monkeys

Eye tracking is a well-established method to assess visual preferences in patients with ASD by analyzing the focus of visual attention^41^. ASD patients often exhibit reduced attention to socially relevant stimuli such as faces^41^. To test whether VPA treatment similarly disrupts visual attention toward social stimuli in non-human primates, we performed eye-tracking analysis following previously published protocols^32^.

On average, the VPA-treated group spent significantly more time on looking at the 80 stimuli of the four categories (541.1 ± 105.4 ms per stimulus for control vs. 984.6 ± 137.8 ms per stimulus for VPA-treated, *P* = 0.036; Fig. 4e, f). When exposed to the “face” stimuli, the two groups of animals showed a similar eye-movement pattern with similar eye-to-mouth ratios (*P* = 0.629; Fig. 4e, g). However, when exposed to the “social & other” images, VPA-treated animals spent significantly more time looking at the nonsocial portion of images (“other”) (759.02 ± 88.52 ms per stimulus) than the control animals did (380.91 ± 109.74 ms per stimulus, *P* = 0.028; Fig. 4h), while the time spent on “social” scenes was comparable between the two groups. The ratio of fixation duration in “social” versus “other” region appeared reduced in treated monkeys than controls, but there was no significant difference between the two groups (*P* = 0.199; Fig. 4e, h). These results were consistent with findings of reduced attention to social cues in autism children^41–43^. Together, the VPA-treated monkeys seemed to pay more attention to nonsocial stimuli than the control did.

## Discussion

Environmental factors during sensitive brain developmental periods have long-term consequences on brain function^44^. In utero exposure to antiepileptic drug VPA is linked to brain disorders in offspring, most notably ASD^10,45^. However, the etiology of environmental factors on neurodevelopment is far from clear, but pivotal for understanding the etiology of ASD without clear genetic causes. In this study, we obtained two aborted monkeys of different sex and different VPA exposure levels and analyzed VPA-induced abnormalities at the molecular and cellular levels. We found that prenatal VPA exposure could disrupt neurogenesis in non-human primates, resulting in reduced numbers of Ki67-positive neuronal precursors and NeuN-positive mature neurons. In utero VPA exposure also altered the expression levels of multiple proteins associated with glutamatergic and GABAergic neurotransmission. Moreover, the phenotype of VPA-treated monkeys appeared to be dose-dependent, as more severe neurogenesis defects and greater decreases in the expression levels of mGluR5, GluN2B, and DCX were observed in the monkey (M2) exposed to a higher dose of VPA, consistent with the known VPA dose-dependent increase in risk for major congenital malformations in human newborns^46^.

Multiple effects of VPA on neural progenitor cell (NPC) proliferation and neuronal differentiation have been reported in primary cell cultures, including decreased NPC proliferation, increased neuronal differentiation, and inhibition of glial differentiation^47^. Long-term VPA exposure from embryonic day 1 until birth in mice increased the number of NPCs during the early-middle neurogenic period as well as postnatal neuronal production^48^. In contrast, we found thinner Ki67-positive EGL in cerebellum of VPA-exposed monkeys, indicative of fewer neuronal precursors. Our finding is consistent with a report that prenatal VPA exposure at E12.5 to mice causes a decrease in cell proliferation by 7 days and decreased cell density in prefrontal cortex at 8 weeks^38^. In addition, we found fewer neurons, more astrocytes and decreased cell density in full-term fetal brain after prenatal VPA treatment. Glial cells constitute the predominant proportion of human brain cells and play essential roles in neuronal differentiation and metabolism as well as in the regulation of neuroinflammation^49^. Elevated GFAP expression and more GFAP-positive glial cells in VPA-treated monkey PFC suggest alterations in neuronal differentiation, reactive gliosis, or both.

Factors controlling the formation and function of excitatory and inhibitory synapses affect the E/I ratio; altered ratio of E/I is believed to be a possible pathological mechanism underlying ASD^50^. The imbalance of E/I synapses has been reported in VPA rodent models^51^. In this study, western blotting revealed increased expression of the inhibitory synapse marker VGAT but decreased expression of the excitatory marker VGLUT2 in VPA-treated PFC (Fig. 2a, b). The decreased expression of presynaptic VGLUT2 is consistent with the reduced expression of postsynaptic mGluR5 and GluN2B in VPA-treated monkey brain (Fig. 2a, d). In addition to neurogenic defects, the altered E/I synaptic components may also contribute to behavioral abnormalities associated with VPA treatment.

We investigated the changes in transcriptome of monkey brain ~130 days after VPA exposure. Multiple pathways related to neuronal development were altered in VPA-exposed monkey embryos, indicating a long-lasting effect of epigenetic alterations. Gene expression changes right after VPA treatment are attributed mainly to HDAC inhibition^16,17^, while the long-lasting effect of VPA exposure is unknown. We showed that H3K27ac, a marker of active enhancers, was significantly decreased in M1 PFC. Epigenetic modification at enhancers contributes to the specific gene expression programs that determine cell proliferation and differentiation^52^. A VPA rat model displays a disrupted amygdala transcriptome at different time points (P10 and P21), suggesting that VPA disrupts molecular pathways related to autism-like behaviors^17^. Given the dramatic changes at the cellular level revealed by immunostaining, it is not surprising that a vast number of DEGs (1722) were identified upon VPA treatment.

Clinical studies of ASD patients report a defect in engaging attention to socially relevant stimuli. Specifically, individuals with ASD look less at the social scenes and monitor less the play interactions^42,43^. In agreement with the clinical studies, our eye-tracking analysis showed that VPA-treated monkey offspring paid significantly more attention to the non-social scene when presented with “social & other” images (Fig. 4h). VPA-exposed rodents display more pervasive autism-like behavioral deficits, including reduced sociability, stereotypies, and sensory anomalies^53^, while VPA-treated monkeys showed a lower penetrance of autism-like behaviors. The low penetrance of autism-like behaviors in monkeys may attribute to sex effects, as male VPA rats showed substantial social interaction deficits compared to female offspring^54,55^, and only one out of five VPA-treated monkeys reported in this study was male (t5). Individual difference is another explanation for the severe neurogenic defects found in the aborted fetal brain but low penetrance of autism-like behaviors in the five live offspring. Differences in maternal and/or infant metabolism and genetic susceptibility have been reported to affect the teratogenicity of VPA^56^. Children exposed to VPA during pregnancy also demonstrate variable symptom severity^9,10^, supporting the existence of individual difference in drug susceptibility. We suspect that monkeys that are more sensitive to VPA exposure probably failed to develop to term, while the less sensitive individuals survived to adulthood. The apparent variability among VPA exposure animals is consistent with human cases, i.e., a large number of pregnant women were exposed to VPA, but only small percentage of the offspring were abnormal, suggesting different individual susceptibility.

In summary, we found pronounced neurogenesis defects and autism-related behavioral abnormalities in VPA-exposed monkey offspring. VPA-exposed monkeys may offer novel insights into the pathophysiology of VPA-induced developmental defects and behavioral impairments in ASD patients. Studies of VPA treatment in both rodents and non-human primates reveal similarities but also clear and substantial differences in neurological and behavioral outcomes. In utero VPA-treated rodents are a valid model for ASD research, while the value of VPA-exposed monkeys for ASD research requires further testing (such as the prevalence and severity of sex-specific social interaction defects). Nonetheless, this study confirms that VPA has substantial effects on neurogenesis that may account for behavior deficits in primates. Further study on the use of this non-human primate model for ASD is warranted.

## Supplementary information

Supplementray Figure 1

Supplementray Figure 2

Supplementary Legends

Supplementary Tables

Supplementary Table 6

Supplementary Video
