# Supplementary figures and images for: Maternal valproic acid exposure leads to neurogenesis defects and autism-like behaviors in non-human primates
Source: Transl Psychiatry. 2019 Oct 21;9:267. doi: 10.1038/s41398-019-0608-1 (PMC6803711; doi:10.1038/s41398-019-0608-1)

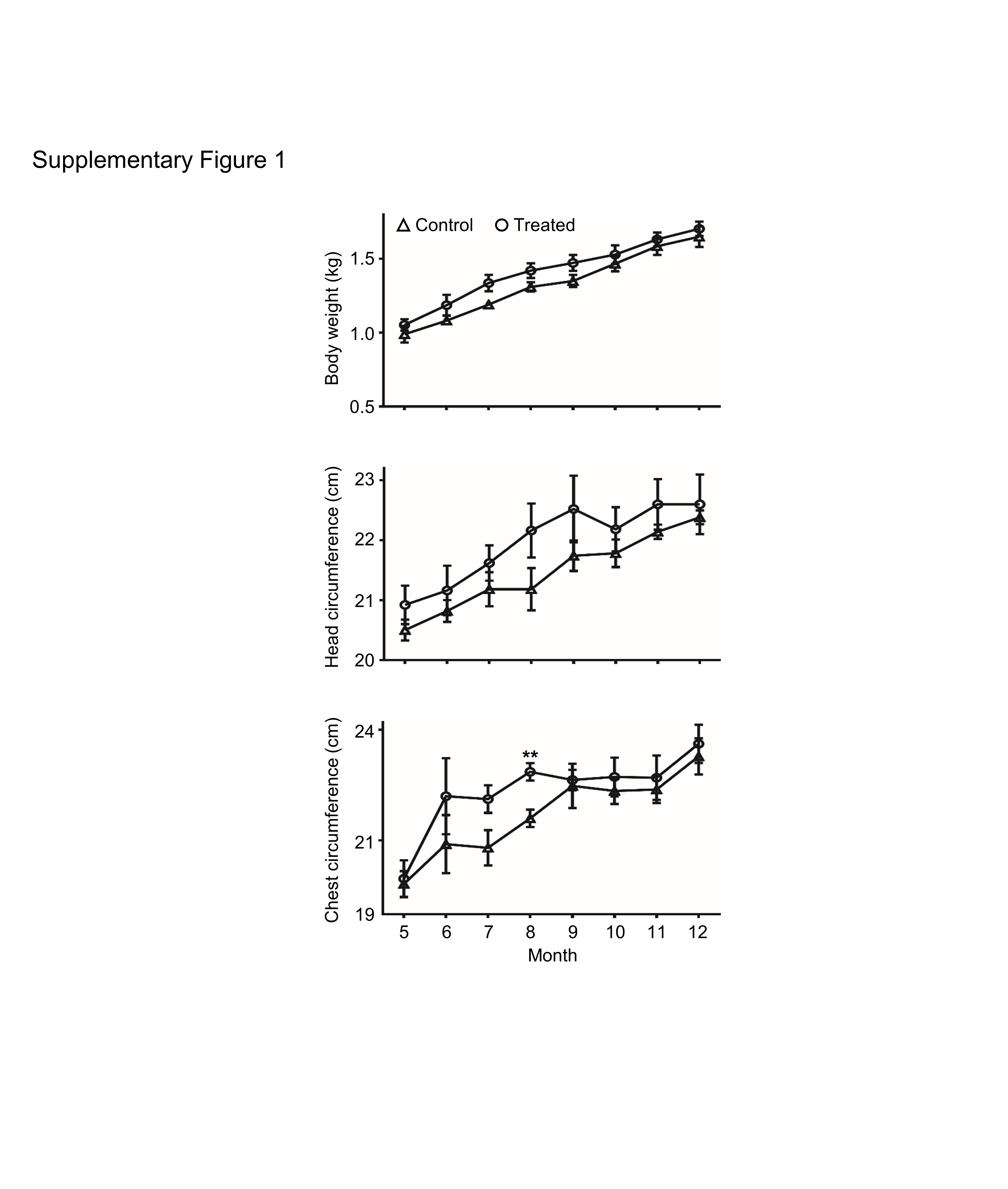

Supplement: Supplementary file 1 — Supplementray Figure 1 [file 41398_2019_608_MOESM1_ESM.tif]

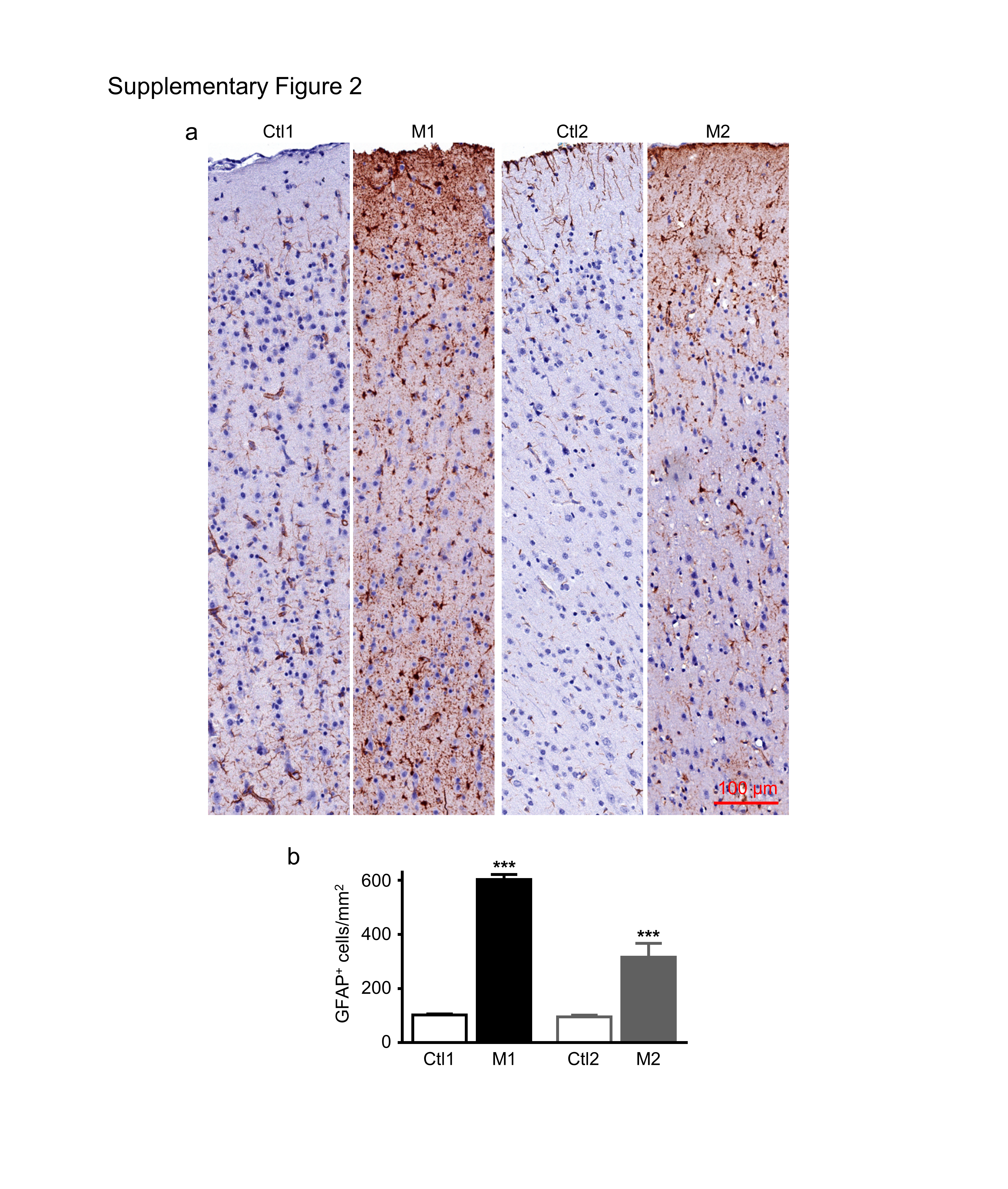

Supplement: Supplementary file 2 — Supplementray Figure 2 [file 41398_2019_608_MOESM2_ESM.tif]
