# Supplementary material for: Maternal valproic acid exposure leads to neurogenesis defects and autism-like behaviors in non-human primates
Source: Transl Psychiatry. 2019 Oct 21;9:267. doi: 10.1038/s41398-019-0608-1 (PMC6803711; doi:10.1038/s41398-019-0608-1)
Supplement: Supplementary file 3 — Supplementary Legends [file 41398_2019_608_MOESM3_ESM.docx]

**Supplementary legends**

**Supplementary Figure 1.** VPA-treated and control monkeys show similar physical development. Body weight, head circumference, and chest circumference are presented for VPA-treated and control monkeys. Error bars represent s.e.m., ***P* < 0.01 by Student’s *t*-test.

**Supplementary Figure 2.** Increased GFAP staining in the PFC of VPA-exposed offspring. (**a**) Immunostaining for GFAP (brown) in the PFC. Nuclei were stained with hematoxylin (blue). (**b**) Quantitative analysis of GFAP-positive cells in the PFC of different individuals. Error bars represent s.e.m., n = 4 slices, ****P* < 0.001 by Student’s *t*-test.

**Supplementary Video. Stereotyped behavior in a VPA-treated monkey offspring.**

VPA-treated monkey t1 has a black collar, while t2 has a blue collar; wild type control ctl1 has a white collar, while ctl2 has a red collar.
