# Supplementary material for: Maternal valproic acid exposure leads to neurogenesis defects and autism-like behaviors in non-human primates
Source: Transl Psychiatry. 2019 Oct 21;9:267. doi: 10.1038/s41398-019-0608-1 (PMC6803711; doi:10.1038/s41398-019-0608-1)
Supplement: Supplementary file 4 — Supplementary Tables [file 41398_2019_608_MOESM4_ESM.docx]

**Supplementary Table 1. List of antibodies**

| **Antibody** | **Source** | **Cat. No.** | **Company** | **WB/IHC Dilution** |
| --- | --- | --- | --- | --- |
| α -tubulin | Mouse | T5168 | Sigma | WB 1:25,000 |
| GluN2B | Rabbit | 4212S | Cell Signaling | WB 1:2,000 |
| mGluR5 | Rabbit | ab5675 | Millipore | WB 1:2,000 |
| Homer1b/c | Rabbit | sc-20807 | Santa Cruz | WB 1:1,000 |
| PSD95 | Rabbit | Ab18258 | Abcam | WB 1:1,000 |
| NeuN | Mouse | Ab104224 | Abcam | WB 1:5,000; IHC 1:1,000 |
| GFAP | Rabbit | Z0334 | Dako | WB 1:20,000 |
| Ki-67 | Mouse | ZM-0166 | ZSGB | IHC 1:100 |
| DCX | Rabbit | 4604 | Cell Signaling | WB 1:1,000 |
| VGAT | Rabbit | AB5062P | Millipore | WB 1:500 |
| VGLUT2 | Mouse | MAB5504 | Millipore | WB 1:2,000 |
| Histone H3 | Rabbit | ab1791 | Abcam | WB 1:10, 000 |
| H3K9ac | Rabbit | 07-352 | Millipore | WB 1:2,000 |
| H3K27ac | Rabbit | ab4729 | Abcam | WB 1:2,000 |

**Supplementary Table 2.** **DEGs confirmed by qRT-PCR**

| *Gene* | Annotation |
| --- | --- |
| *SHANK3* | SH3 and multiple ankyrin repeat domains 3 |
| *SLC1A2* | Solute carrier family 1 (glial high affinity glutamate transporter), member 2 |
| *PAX6* | Paired box 6 |
| *DBI* | Diazepam binding inhibitor, Acyl-CoA binding protein |
| *SHANK1* | SH3 and multiple ankyrin repeat domains 1 |
| *GRIN2C* | Glutamate receptor, ionotropic, N-methyl D-aspartate 2C |
| *SYNDIG1L* | Synapse differentiation inducing 1 like |
| *BMP7* | Bone morphogenetic protein 7 |
| *S100A8* | S100 calcium binding protein A8 |

Note: the first five genes are autism related and listed in the order of relevance to autism (highest in the top) based on SFARI database [(https://gene.sfari.org/).](file:///C:\Users\YQ%20Zhang\AppData\Roaming\Foxmail7\Temp-4788-20180506085151\(https:\gene.sfari.org\))

**Supplementary Table 3. Primers for qRT-PCR analysis of target genes**

| **Gene** | **Primer sequence (5’-3’)** | **Size (bp)** |
| --- | --- | --- |
| *SHANK3* | TGCCACATACACACTCGGTC | 95 |
|  | GGAGGTGAAGCGGAACTGAC |  |
| *SLC1A2* | TGTGGGTGACTCTTTTGGGG | 78 |
|  | CGATGCTGGGAGTCAATGGT |  |
| *PAX6* | CCAGATGTGTTTGCCCGAGA | 138 |
|  | GTTGCTGGCCTGTCTTCTCT |  |
| *DBI* | ACTAGGCCATGTGTTTCTCCT | 118 |
|  | CGTATGGTGAGCAGCCTTGA |  |
| *SHANK1* | TGATGCCCCAAGCGATTACA | 75 |
|  | AACCCAAACCCCTCACTGTC |  |
| *GRIN2C* | GGGTGGTCAAATTCTCCTACGAC | 102 |
|  | TGTAGTACACCTCCCCAATCAT |  |
| *SYNDIG1L* | GAAGGAATGGCCTGCTGAGT | 142 |
|  | GCTACACCCCAGCCTACAAA |  |
| *BMP7* | CAGCCTCTGGTTAGCATCGT | 126 |
|  | GCCATCCTGAACGAGGGATT |  |
| *S100A8* | GGTGCAGATGCCTGGTTCAA | 95 |
|  | GCCACGCCCATCTTTATCAC |  |
| *GAPDH* | TCTCTGCTCCTCCTGTTCGAG | 105 |
|  | GACCAAATCCGTTGACTCCGAC |  |

**Supplementary Table 4. Information for social behavior assay and eye-tracking experiment.**

| **Animal** | **Gender** | **Date of birth (y.m.d)** | **Video-recording dates (y.m.d)** | **Eye-tracking dates (y.m.d/d)*** |
| --- | --- | --- | --- | --- |
| ctl1 | F | 2015.12.16 | Cage 1  2017.9.14-21 | 2017.11.7/9 |
| t1 | F | 2015.12.08 |  | 2017.11.20/21 |
| ctl2 | F | 2016.1.24 |  | 2017.10.30/31 |
| t2 | F | 2016.1.23 |  | 2017.12.22/25 |
| ctl3 | F | 2016.3.5 | Cage 2  2017.9.11-20 | 2018.2.6/7 |
| t3 | F | 2016.3.3 |  | 2017.11.14/15 |
| ctl4 | F | 2016.4.25 |  | 2017.11.29/30 |
| t4 | F | 2016.4.23 |  | 2017.11.23/28 |
| ctl5 | M | 2016.2.19 | Cage 3  2017.9.8-13 | 2018.2.2/5 |
| t5 | M | 2016.2.18 |  | 2017.12.27/28 |
| ctl2 | F | 2016.1.24 |  | 2017.10.30/31 |
| t2 | F | 2016.1.23 |  | 2017.12.22/25 |

* indicates two specific days on which eye-tracking experiments were carried out.

**Supplementary Table 5. Behavioral ethogram**

| **Behavior** | **Description** | **Ref.** |
| --- | --- | --- |
| Active social behavior | Sitting within other monkey’s arms;  Grooming for others. | 1 |
| Passive social behavior | Receiving active social behaviors from other monkeys. | 2 |
| Stereotypies | At least two consecutive backflips;  Repetitive, undirected walking with the same path for at least 3 seconds;  Repetitive twirling for at least two rounds;  Head-twist when turning at corners. | 3 |
| Exploratory behavior | Sniffing, searching, and manipulating the cage lock non-repetitively. | 4 |

Reference:

1. Bliss-Moreau, E., Moadab, G., Bauman, M.D. & Amaral, D.G. The impact of early amygdala damage on juvenile rhesus macaque social behavior. *J. Cogn. Neurosci.* **25**, 2124–2140 (2013).
2. Tu, Z. et al. CRISPR/Cas9-mediated disruption of *SHANK3* in monkey leads to drug-treatable autism-like symptoms. *Hum. Mol. Genet.* **28**, 561–571 (2019).
3. Bauman, M.D., Toscano, J.E., Babineau, B.A., Mason, W.A. & Amaral, D.G. Emergence of stereotypies in juvenile monkeys (*Macaca mulatta*) with neonatal amygdala or hippocampus lesions. *Behav. Neurosci.* **122**, 1005–1015 (2008).
4. Camus, S.M., Blois-Heulin, C., Li, Q., Hausberger, M. & Bezard, E. Behavioural profiles in captive-bred cynomolgus macaques: towards monkey models of mental disorders? *PLoS ONE* **8**, e62141 (2013).

**Supplementary Table 6. Differentially expressed genes identified by RNA-seq.**
